# *hOGG1* Ser326Cys Polymorphism and Risk of Hepatocellular Carcinoma among East Asians: A Meta-Analysis

**DOI:** 10.1371/journal.pone.0060178

**Published:** 2013-04-05

**Authors:** Wenjun Wang, Shuangsuo Dang, Yaping Li, Mingzhu Sun, Xiaoli Jia, Rui Wang, Jingkun Liu

**Affiliations:** Department of Infectious Diseases, Second Affiliated Hospital of Medical School, Xi’an Jiaotong University, Xi’an, China; Medical University Graz, Austria

## Abstract

**Background:**

The *hOGG1* gene encodes a DNA glycosylase enzyme responsible for DNA repair. The Ser326Cys polymorphism in this gene may influence its repair ability and thus plays a role in carcinogenesis. Several case-control studies have been conducted on this polymorphism and its relationship with the risk of hepatocellular carcinoma (HCC) among East Asians. However, their results are inconsistent.

**Methods:**

We performed a meta-analysis of published case-control studies assessing the association of the *hOGG1* Ser326Cys polymorphism with HCC risk among East Asians. PubMed, EMBASE, SCI, BIOSIS, CNKI and WanFang databases were searched. A random-effect model was used to calculate odds ratios (ORs) and 95% confidence intervals (95% CIs). Analyses were conducted for additive, dominant and recessive genetic models.

**Results:**

Eight studies were identified involving 2369 cases and 2442 controls assessing the association of the *hOGG1* Ser326Cys polymorphism with HCC risk among East Asians. Applying a dominant genetic model, only in the Chinese population, the Cys allele was significantly associated with increased risk of HCC (OR 1.56, 95% CI 1.12–2.17). However, two studies influenced this finding according to sensitivity analysis. Furthermore, considerable heterogeneity and bias existed among Chinese studies.

**Conclusion:**

There is limited evidence to support that the *hOGG1* Ser326Cys polymorphism is associated with HCC risk among East Asians. Well-designed and large-sized studies are required to determine this relationship.

## Introduction

Hepatocellular carcinoma (HCC) is the sixth most prevalent cancer and the third most frequent cause of cancer-related death worldwide [1]. The highest prevalence of HCC is in East Asia due to the high prevalence of chronic infection with hepatitis B virus (HBV) [2]. Other well-established risk factors for HCC include chronic infection with hepatitis C virus (HCV), exposure to aflatoxin B1, male gender, drinking, smoking, non-alcoholic fatty liver disease and diabetes [3], [4], [5], [6], [7]. In the past two decades, more and more GWAS (genome-wide association studies) and other gene-disease association studies have found that some variants in human genes are associated with HCC, indicating that genetic background also plays a role in hepatocellular carcinogenesis.

Human 8-hydroxyguanine glycosylase 1 (hOGG1) is a DNA glycosylase enzyme responsible for the excision of 8-oxoguanine, a mutagenic base byproduct which occurs as a result of exposure to reactive oxygen [8]. The *hOGG1* gene, located on chromosome 3p26.2, is composed of eight exons and seven introns. Polymorphisms in this gene may alter glycosylase function and an individual’s ability to repair damaged DNA, possibly resulting in genetic instability that can foster carcinogenesis [8]. Among many polymorphisms identified in the *hOGG1* gene, much interest has been focused on the Ser326Cys (C>G) polymorphism (rs1052133). It is in exon 7 of the *hOGG1* gene, which takes the form of a single amino acid substitution, from serine to cysteine at condon 326. Although the evidence is inconclusive that this functional polymorphic variation influences the activity of hOGG1 [8], many epidemiologic studies have been conducted to examine its relationship with cancer risk.

In the past years, several studies have investigated the association of the *hOGG1* Ser326Cys polymorphism with HCC risk among East Asians [9], [10], [11], [12], [13], [14], [15], [16]. Some found that the Cys allele was associated with increased risk of HCC [13], [16]. However, the others found no association [9], [10], [11], [12], [14], [15]. Such inconsistency could be due partly to insufficient power, the small effect of the Ser326Cys polymorphism on HCC risk and false-positive results. We therefore performed a meta-analysis of published studies to investigate whether the Ser326Cys polymorphism has an effect on HCC susceptibility.

## Methods

### Searching

**Figure 1:**
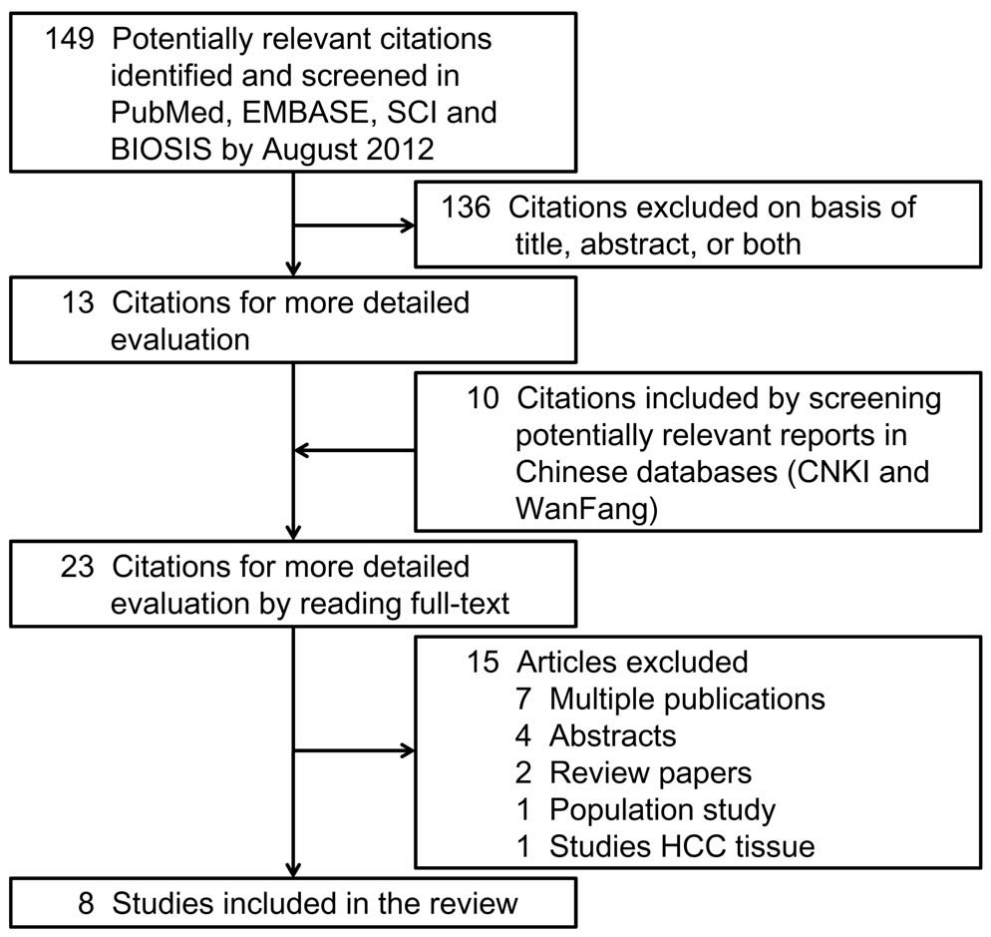
Flow diagram of the study selection process.

We searched PubMed, EMBASE, ISI Science Citation Index, BIOSIS, and Chinese electronic databases including CNKI and WanFang. The last search update was performed in August 2012. The search strategy was based on combinations of terms for *hOGG1* and HCC (see Methods S1) without language restriction. References of retrieved reviews and articles for more detailed evaluation after reading the titles and abstracts were also screened. All case-control designed studies were considered eligible if they aimed to investigate the relation between the *hOGG1* Ser326Cys polymorphism and HCC risk. Conference abstracts and review articles were excluded. Figure 1 describes the study selection process that led to the final 8 studies in this meta-analysis.

Two of the authors (WW & YL) independently identified and reviewed each relevant study. Disagreements were reconciled through group discussion. When more than one record was identified for the same study population, we included the most recent publication or population including more information.

### Data Abstraction

Following the Meta-analysis Of Observational Studies in Epidemiology (MOOSE) statement for reporting meta-analyses of observational studies [17], we used a standardized reporting form to abstract data from each included study. For each study, the following information was extracted independently by two investigators (WW & YL): the first author’s name, year of publication, study design, ethnicity, definition and numbers of cases and controls, confounding factors by matching or adjustment, genotyping method, frequency of genotypes, odds ratios (ORs) and 95% confidence intervals (95% CIs) for HCC associated with the *hOGG1* Ser326Cys polymorphism, and consistency of genotype frequencies with Hardy-Weinberg equilibrium (HWE) in control subjects.

### Statistical Analysis

We referred to a previous study to perform statistical analysis [18]. ORs with 95% CIs were calculated to assess the strength of the association between the *hOGG1* Ser326Cys polymorphism and HCC risk. The association was examined under three genetic models: the additive model (Cys/Cys vs. Ser/Ser), the dominant model (Ser/Cys+Cys/Cys vs. Ser/Ser) and the recessive model (Cys/Cys vs. Ser/Cys+Ser/Ser). HWE was tested using the chi-squared test and it was considered statistically significant when the *P* value is less than 0.05. Sensitivity analyses were carried out using the one-study remove approach to assess the impact of each study on the combined effect.

Heterogeneity assumption was checked by the *I^2^* statistic and a chi-square based *Q* test. A *P* value of more than 0.05 for the *Q* test indicated a lack of heterogeneity among the studies, so the summary OR estimate of each study was calculated by the fixed-effect model (the Mantel-Haenszel method) [19]. Otherwise, the random-effect model (DerSimonian and Laird method) was used [20]. Egger’s test and Begg’s graphical methods were used to provide diagnosis of the potential publication bias [21]. All statistical analyses were performed with STATA software (version 10.0, StataCorp LP, College Station, Texas, USA) and RevMan software (version 5.1, Cochrane Collaboration). This meta-analysis has a protocol (see Methods S2). The performance and report of this meta-analysis comply with PRISMA Statement (see Methods S3).

## Results

### Eligible Studies

There were 8 studies identified on the *hOGG1* Ser326Cys polymorphism and HCC susceptibility (Figure 1) [9], [10], [11], [12], [13], [14], [15], [16]. These 8 independent studies were published from 2004 to 2012 with 5 in Chinese language [9], [11], [12], [13], [14] and 3 in English [10], [15], [16]. In total, 2369 cases and 2442 controls were included. Table 1 shows the detailed characteristics of each study. All studies were conducted in East Asia, an area with high incidence of HCC. 6 studied Chinese population [9], [11], [12], [13], [14], [16], 1 studied Japanese population [10], and 1 studied Korean population [15]. 1 study did not supply age and sex information [9]. In the other 7 studies supplying this information, all but one had matched age and sex in case and control groups [10]. For control subjects, 4 studies recruited among hospital patients with HCC-unrelated diseases [9], [10], [11], [13], 2 studies among people with a comparable HBV background [12], [16], 1 study among people with chronic liver diseases (97% were infected with HCV and/or HBV) [10], 1 study among HBV chronically infected people [15], and 1 study among healthy people [14]. Only 1 study extracted DNA from formalin-fixed or paraffin-embedded liver tissues [9]. The others extracted from blood samples. All studies but one were consistent with HWE (*P*<0.001) [13].

**Table 1:** Characteristics of studies included in this meta-analysis.

Study	Ethnicity	Screening of controls	Genotype method	Case			Control			Confounding factors adjusted or stratified	Cys(%)	HWE(*P* value)
				Male (%)	Age(mean, sd)	Cys-Cys/Cys-Ser/Ser-Ser	Male (%)	Age(mean, sd)	Cys-Cys/Cys-Ser/Ser-Ser			
Zhu, 2004	Chinese	Hospital	PCR-RFLP	NA	NA	57/99/37	NA	NA	50/62/22	Sex, age, smoking, drinking, HBV, HCV, family history of HCC	60.4	>0.7
Sakamoto, 2006	Japanese	Hospital	PCR-CTPP	67%	69	43/110/56	65%	61	79/123/73	Sex, age, smoking, drinking, HBV, HCV	51.1	>0.05
		CLD†					54%	61	100/176/105		49.3	>0.1
Zhang, 2006‡	Chinese	Hospital	Sequencing	84%	49.9±18.0	18/38/30	82%	49.9±17.0	13/35/42	HBV	33.9	>0.2
Wang, 2008	Chinese	HBVcomparable§	Taqman	84%	50.2±11.3	52/92/31	matched	matched	34/58/27	Sex, age, smoking, drinking, HBV, HCV, family history of HCC	52.9	>0.8
Ji, 2011	Chinese	Hospital	Taqman	79%	47.8±11.1	103/40/357	74%	48.9±11.2	29/51/427	Drinking, HBV	10.7	<0.001
Tang, 2011	Chinese	Healthy	PCR-RFLP	100%	matched	47/82/21	100%	matched	56/72/22	None	61.3	>0.8
Jung, 2012*	Korean	HBV	SNPstream	82%	53.3±8.3	207/343/156	83%	52.6	116/181/89	Sex, age	53.3	>0.2
Yuan, 2012	Chinese	HBVcomparable§	PCR-RFLP	76%	52.1±7.6	66/217/67	75%	51.1±9.2	50/206/144	Sex, age, drinking, HBV, family history of HCC	38.3	>0.05

HWE, Hardy-Weinberg equilibrium; PCR, polymerase chain reaction; RFLP, restriction fragment length polymorphism; HBV, hepatitis B virus; HCV, hepatitis C virus; HCC, hepatocellular carcinoma; CLD, chronic liver diseases; CTPP, confronting two-pair primers; NA, not available.

† In this control group, 97% of subjects were infected with HCV and/or HBV.

‡ Sex and age information was from 91 case subjects and 91 control subjects.

§ Case group and control group had comparable background of HBV infection.

* Sex and age information was from 708 case subjects and 388 control subjects.

### Meta-analysis Results

**Figure 2:**
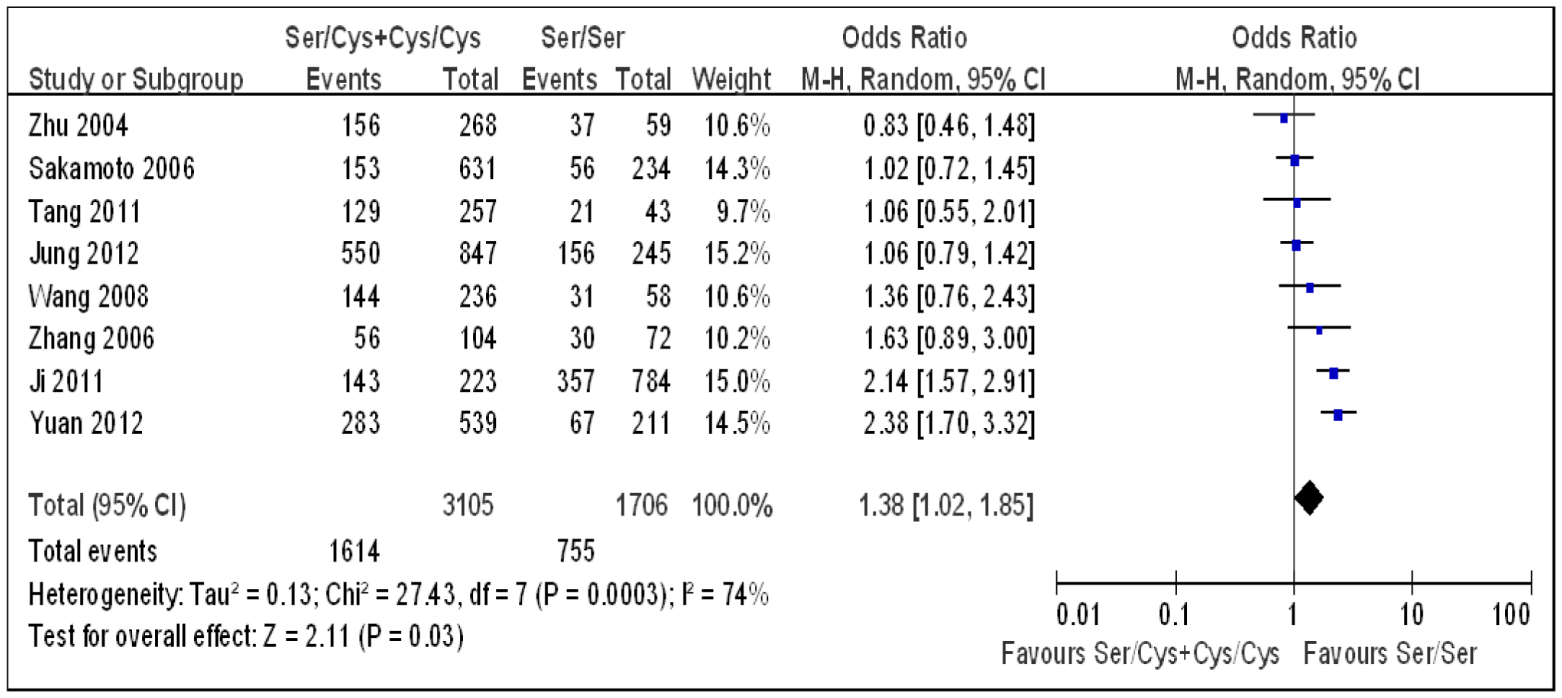
Forest plots for the *hOGG1* Ser326Cys polymorphism and risk of hepatocellular carcinoma using the dominant genetic model (Ser/Cys+Cys/Cys vs. Ser/Ser). The squares and horizontal lines correspond to the study specific odds ratios and 95% confidence intervals. The diamond represents the summary odds ratio and 95% confidence interval.

**Figure 3:**
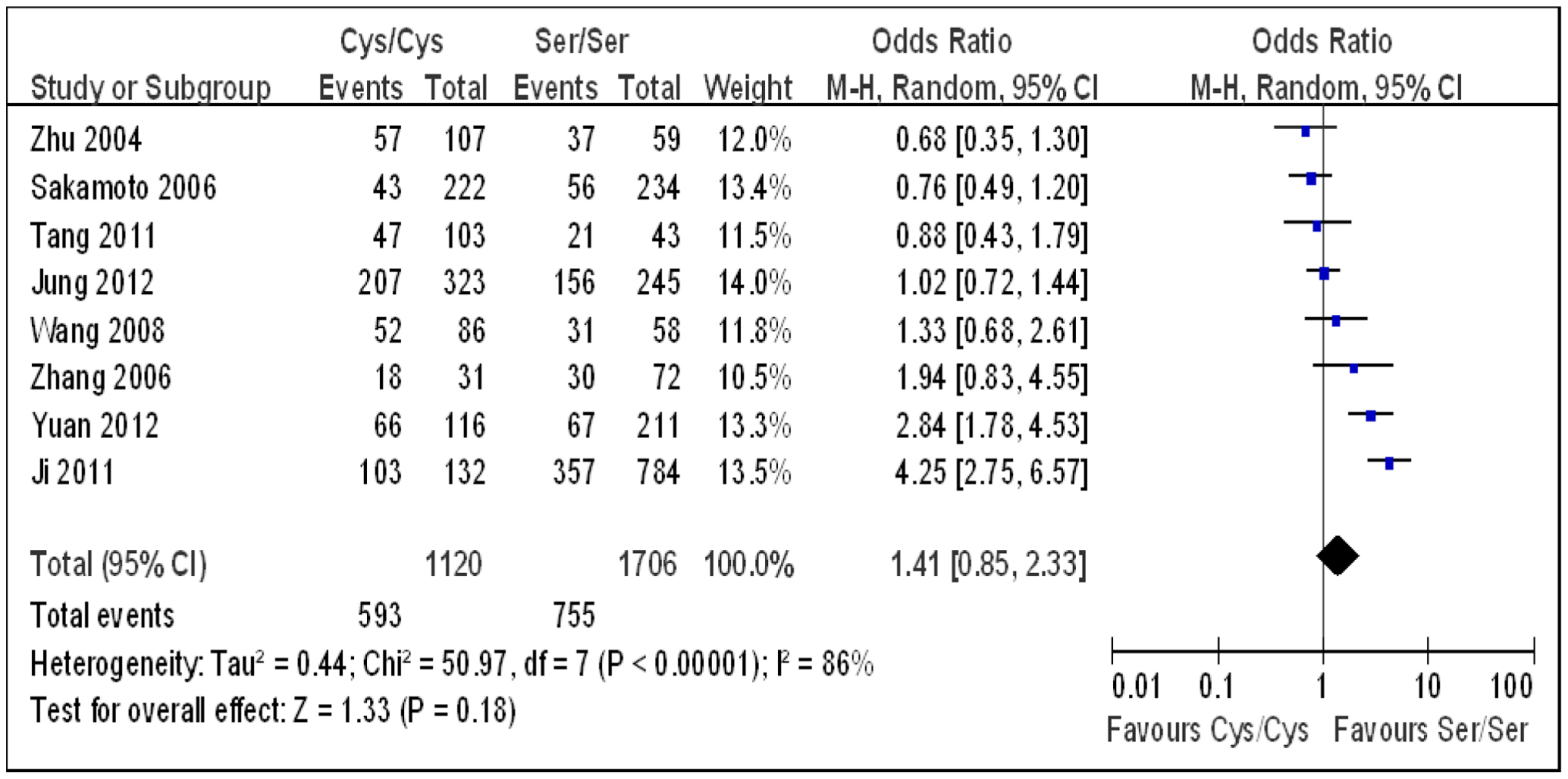
Forest plots for the *hOGG1* Ser326Cys polymorphism and risk of hepatocellular carcinoma using the additive genetic model (Cys/Cys vs. Ser/Ser). The squares and horizontal lines correspond to the study specific odds ratios and 95% confidence intervals. The diamond represents the summary odds ratio and 95% confidence interval.

**Figure 4:**
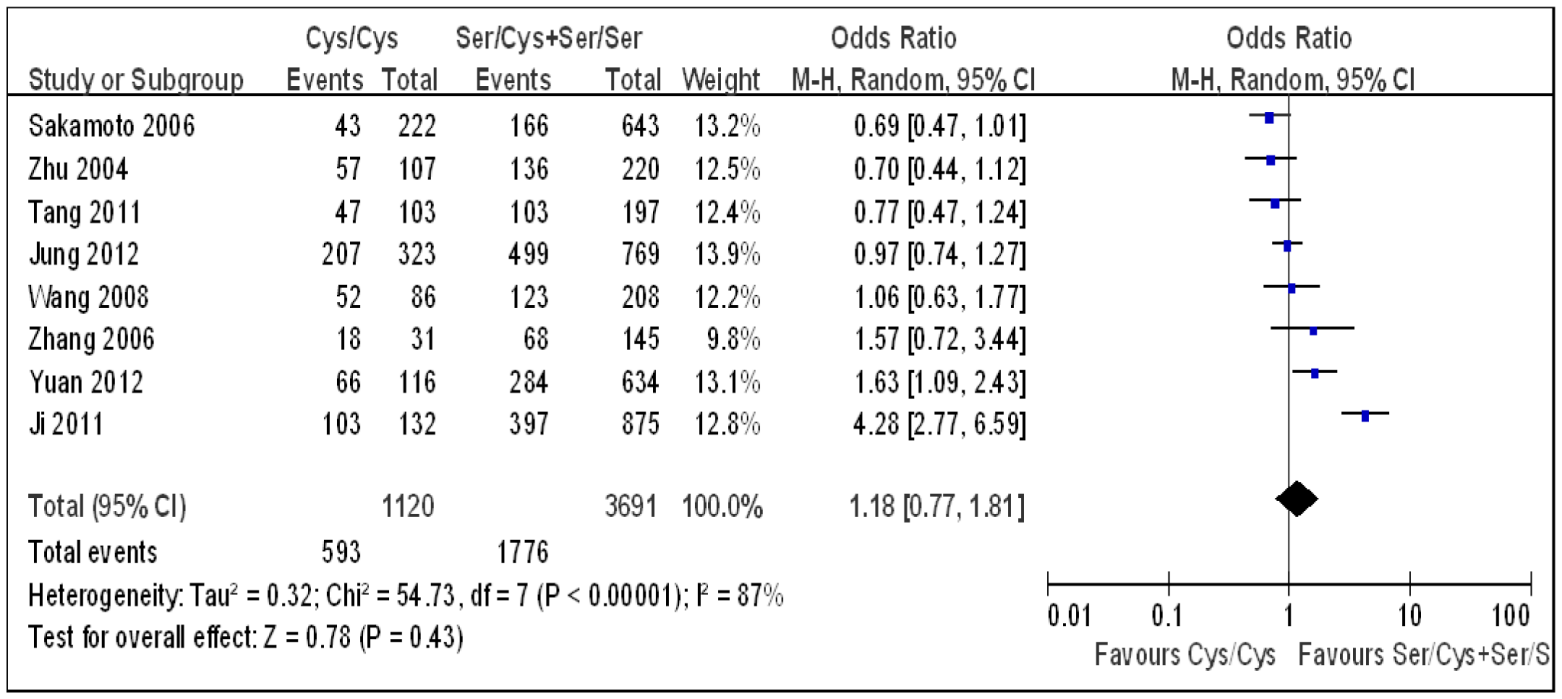
Forest plots for the *hOGG1* Ser326Cys polymorphism and risk of hepatocellular carcinoma using the recessive genetic model (Cys/Cys vs. Ser/Cys+Ser/Ser). The squares and horizontal lines correspond to the study specific odds ratios and 95% confidence intervals. The diamond represents the summary odds ratio and 95% confidence interval.

Under each genetic model, heterogeneity assessment showed significant variation across studies. Therefore, a random-effect model was used to analyze the summary ORs. As Sakamoto *et al.* used two control groups (hospital controls and chronic liver disease controls) [10], first we combined the two groups to carry out the overall analysis. Combining all studies, a significant positive association between the *hOGG1* Ser326Cys polymorphism and HCC risk was observed under the dominant genetic model (OR 1.38, 95% CI 1.02–1.85; Table 2, Figure 2). No significant association was found under the additive (OR 1.41, 95% CI 0.85–2.33; Table 2, Figure 3) and the recessive models (OR 1.18, 95% CI 0.77–1.81; Table 2, Figure 4). Then, we used each control group in the study by Sakamoto *et al.* to carry out the overall analysis. The results hardly changed (data not shown). As the *hOGG1* Ser326Cys polymorphism in control subjects did not fulfill HWE in the study by Ji *et al.* [13], we excluded this study and repeated the above analyses. No significant association was found under any genetic models (Table 2). Sensitivity analyses confirmed that several other studies also contributed to the observed significant association between the *hOGG1* Ser326Cys polymorphism and HCC risk under the dominant genetic model (Table 3) [11], [12], [16].

**Table 2:** Meta-analysis of published association between the *hOGG1* Ser326Cys polymorphism and HCC risk.

Genetic model (No. of studies)	Case	Control	Analysis model	Summary OR (95% CI)	*P* †	*P* ‡
Overall§ (8)						
Additive model	1348	1478	Random	1.41 (0.85–2.33)	<0.00001	0.86
Dominant model	2369	2442	Random	**1.38 (1.02–1.85)**	<0.001	0.52
Recessive model	2369	2442	Random	1.18 (0.77–1.81)	<0.00001	0.69
Chinese population (6)						
Additive model	886	916	Random	1.67 (0.91–3.08)	<0.0001	0.08
Dominant model	1454	1400	Random	**1.56 (1.12–2.17)**	0.01	**0.03**
Recessive model	1454	1400	Random	1.36 (0.76–2.42)	<0.00001	0.72
Consistent with HWE§ (7)						
Additive model	888	1022	Random	1.18 (0.79–1.77)	<0.001	0.87
Dominant model	1869	1935	Random	1.27 (0.94–1.73)	0.003	0.80
Recessive model	1869	1935	Random	0.96 (0.75–1.24)	0.03	0.74
HBV/HCV comparable control* (4)						
Additive model	678	665	Random	1.32 (0.76–2.30)	0.001	0.77
Dominant model	1440	1286	Random	1.37 (0.90–2.11)	0.002	0.97
Recessive model	1440	1286	Random	1.04 (0.76–1.43)	0.04	0.84
Hospital control# (4)						
Additive model	701	735	Random	1.42 (0.52–3.89)	<0.00001	0.60
Dominant model	988	1006	Random	1.33 (0.82–2.14)	0.004	0.38
Recessive model	988	1006	Random	1.32 (0.49–3.51)	<0.00001	0.97

OR, odds ratio; CI, confidence interval.

† *P* value for the *Q* test.

‡ *P* value for Egger’s test.

§ In the study by Sakamoto, subjects from the hospital control and the CLD (chronic liver diseases) control were pooled together.

* Including studies by Sakamoto, Wang, Jung and Yuan. In the study by Sakamoto, data from the CLD (chronic liver diseases) group was used.

# In the study by Sakamoto, data from the hospital group was used.

**Table 3:** Sensitivity analysis using the one-study remove approach.

Study omitted	Additive model		Dominant model		Recessive model	
	OR	95% CI	OR	95% CI	OR	95% CI
Zhu, 2004	1.55	0.91–2.66	1.46	1.08–1.98	1.28	0.80–2.04
Sakamoto, 2006	1.55	0.90–2.66	1.44	1.05–2.00	1.29	0.81–2.05
Zhang, 2006	1.35	0.78–2.35	1.35	0.97–1.86	1.15	0.73–1.82
Wang, 2008	1.42	0.81–2.49	1.37	0.99–1.91	1.20	0.75–1.95
Ji, 2011	1.18	0.79–1.77	1.27	0.94–1.73	0.96	0.75–1.24
Tang, 2011	1.50	0.86–2.59	1.41	1.03–1.95	1.26	0.79–2.02
Jung, 2012	1.48	0.82–2.68	1.44	1.04–2.00	1.23	0.72–2.07
Yuan, 2012	1.26	0.74–2.17	1.26	0.95–1.67	1.13	0.70–1.83
Combined	1.41	0.85–2.33	1.38	1.02–1.85	1.18	0.77–1.81

OR, odds ratio; CI, confidence interval.

Subgroup analyses were performed by dividing studies into groups according to ethnicity and source of controls. Only in the Chinese population, significant association between the *hOGG1* Ser326Cys polymorphism and HCC risk was observed under the dominant genetic model (OR 1.56, 95% CI 1.12–2.17; Table 2). However, this association was lost if the study by Ji *et al.* or by Yuan *et al.* was removed (OR 1.41, 95% CI 0.93–2.14; OR 1.39, 95% CI 0.96–2.02; respectively) [13], [16].

Begg’s funnel plot and Egger’s test were performed to assess the publication bias of the literatures. The results indicated that bias may exist among Chinese studies (*P*_Egger’s test_ = 0.03; Table 2).

## Discussion

The association between the *hOGG1* Ser326Cys polymorphism and HCC risk was not clear due to inconsistent data generated by a range of independent studies. Therefore we performed a meta-analysis of published studies to clarify the inconsistency and to establish a comprehensive picture of this gene-disease association. All studies included in this analysis were conducted in East Asia, an area with high prevalence of HCC. By pooling 8 studies with 2369 cases and 2442 controls, our meta-analysis showed a statistically significant but very weak association between the *hOGG1* Ser326Cys polymorphism and HCC risk when applying a dominant genetic model. Further subgroup analyses revealed that this association only existed in the Chinese population. Other subgroup analyses excluding study not fulfilling HWE or regarding the source of controls did not produce any significant findings. In addition, considerable heterogeneity was detected across studies. And the heterogeneity cannot be fully explained by ethnicity, source of controls and whether fulfilling HWE or not. The study by Yuan et al. is the main source of heterogeneity [16]. This study, with a relatively large sample size (350 cases and 400 controls), studied Chinese population and after adjusting confounding factors exhibited an odds ratio of 2.38 (95% CI 1.80–3.14) [16], indicating a moderate association.

The significant positive findings from meta-analyses are not robust because they are sensitive to four studies in the overall analysis [11], [12], [13], [16] and to two studies in the Chinese subgroup analysis [13], [16] according to the leave-one-out sensitivity analysis. The Egger test suggested the existence of bias among Chinese studies. This may be due to reporting bias, other biases or genuine heterogeneity, and it is difficult to determine which is the case [22]. Taken together, there is limited evidence to support the association between the *hOGG1* Ser326Cys polymorphism and HCC risk.

8-oxoguanine is one of the most common DNA lesions resulting from reactive oxygen species [23]. It has the ability to pair with adenine instead of cytosine during DNA replication, and therefore plays a role in carcinogenesis [24]. In human, hOGG1 is responsible for the repair of 8-oxoguanine. The conduction of studies to examine the *hOGG1* Ser326Cys polymorphism and cancer risk, is based on the notion that this polymorphism may influence the enzyme activity of hOGG1 and thus influence the process of carcinogenesis through 8-oxoguanine. Some studies suggested that the 326Cys allele confers decreased ability to repair 8-oxoguanine [25], [26], [27]. Other studies, however, found no difference in activity by the *hOGG1* Ser326Cys polymorphism [28], [29], [30], [31], [32], [33], [34], [35]. So, whether the *hOGG1* Ser326Cys polymorphism has an impact on the repair of 8-oxoguanine is inconclusive.

High levels of 8-oxogudanine are found in HCC patients (liver tissue: adjacent nontumor tissue>tumor tissue>chronic viral hepatitis>control) and are closely associated with inflammatory infiltration [36], [37]. Peng *et al.* found that levels of 8-oxogudanine were high and levels of hOGG1 were low in peripheral leukocytes from adolescents in a high risk region for HCC in China. Individuals with the 326Ser allele rather than the 326Cys allele had a significantly higher concentration of leukocyte 8-oxogudanine level [38]. Tang *et al.* studied the urea 8-oxogudanine level in HCC patients, and did not find a relationship with the *hOGG1* Ser326Cys polymorphism [14]. However, these studies had a relatively small sample size, and were unable to control for other factors that may affect 8-oxogudanine levels. Together with our meta-analysis, there is lacked evidence to support a link between the *hOGG1* Ser326Cys polymorphism and HCC development.

Our meta-analysis has several limitations. Firstly, only 8 published studies were included, thus the meta-analysis was restricted to a relatively small population. All the 8 studies studied East Asian population, a population with high-HCC risk. So, our findings are not suitable for other populations, especially in Caucasian population which has a low-HCC risk. Recently, one study involving Caucasian population failed to find any association between the *hOGG1* Ser326Cys polymorphism and HCC risk [39]. As it was reported as meeting abstract and further information was not available by contacting the authors, it was not included in our meta-analysis. Secondly, polymorphisms that affect disease susceptibility may do so only in the presence of a relevant exposure; in the case of *hOGG1*, these include hepatitis virus, smoking, alcohol consumption, meat intake, and other factors that are thought to induce DNA damage [8]. However, only three included studies reported the *hOGG1* Ser326Cys polymorphism in populations exposed to some of the above factors [10], [11], [16]. And the numbers of involved subjects are too small to draw a conclusion. Thirdly, the heterogeneity of control groups should be noticed. In most meta-analysis, controls are roughly divided into hospital controls and population controls. Considering the overwhelming impact of HBV and HCV on HCC development, we divided controls into hospital controls, healthy controls and HBV/HCV background comparable controls. However, hospital controls are from patients with different diseases, patients with HBV or HCV have various statuses such as inactive carrier for long years and liver cirrhosis. These conditions are different in the included studies, and thus may exaggerate or underestimate the real effect of the *hOGG1* Ser326Cys polymorphism on HCC risk. Fourthly, like most meta-analysis, this study is based on unadjusted estimates, while a more precise analysis might be conducted if individual data were available, which could allow for an adjusted estimate by confounding factors. At last, quality of reporting is low in most included studies, although lack of reporting should not be assumed to imply poor quality of a study [22].

We reviewed full-text and supplementary data of GWAS studies of HCC identified in the Catalogue of Published Genome-Wide Association Studies (http://www.genome.gov/gwastudies/). The *hOGG1* Ser326Cys polymorphism has not been highlighted in these studies.

In conclusion, there is limited evidence to support that the *hOGG1* Ser326Cys polymorphism is associated with HCC risk among East Asian populations. Well-designed and large-sized studies are required to determine this relationship.

## Supporting Information

Methods S1 **Search strategies.**
(DOCX)

Methods S2 **Protocol for this meta-analysis.**
(DOCX)

Methods S3 **Checklist to confirm compliance with PRISMA guidelines for systematic reviews and meta-analyses.**
(DOCX)
