# Supplementary material for: hOGG1 Ser326Cys Polymorphism and Risk of Hepatocellular Carcinoma among East Asians: A Meta-Analysis
Source: PLoS One. 2013 Apr 5;8(4):e60178. doi: 10.1371/journal.pone.0060178 (PMC3618171; doi:10.1371/journal.pone.0060178)
Supplement: Methods S1 — Search strategies. (DOCX) [file pone.0060178.s001.docx]

MEDLINE

#1 "liver neoplasms"[Mesh]

#2 (hepatocellular OR hepato-cellular OR liver OR hepatic) AND (carcinoma* OR cancer OR tumor OR tumour OR neoplas* OR malign*)

#3 hepatoma* OR hcc

#4 #1 OR #2 OR #3

#5 “8-Oxoguanine glycosylase” OR “8-oxoguanine DNA N-glycosylase” OR OGG1 OR HOGG1

#6 #4 AND #5

EMBASE

#1 "liver cancer"/exp

#2 (hepatocellular OR hepato-cellular OR liver OR hepatic).ti,ab.

#3 (carcinoma* OR cancer* OR tumo* OR neoplas* OR malign*).ti,ab.

#4 #2 AND #3

#5 #1 OR #4

#6 “8-Oxoguanine glycosylase” /

#7 “8-oxoguanine DNA N-glycosylase”/

#8 OGG1/

#9 HOGG1/

#10 #6 OR #7 OR #8 OR #9

#11 35 AND #10

Web of Science & Biosis:

#1 TS=((hepatocellular OR hepato-cellular OR liver OR hepatic) AND (carcinoma* OR cancer* OR tumo* OR neoplas* OR malign*))

#2 TS=(hepatoma* OR hcc)

#3 #1 OR #2

#4 “8-Oxoguanine glycosylase” /

#5 “8-oxoguanine DNA N-glycosylase”/

#6 OGG1/

#7 HOGG1/

#8 #4 OR #5 OR #6 OR #7

#9 #3 AND #8

CNKI/WanFang

(肝癌 OR 肝细胞肝癌) AND (OGG1 OR HOGG1 OR 羟脱氧鸟苷)
