# Supplementary material for: hOGG1 Ser326Cys Polymorphism and Risk of Hepatocellular Carcinoma among East Asians: A Meta-Analysis
Source: PLoS One. 2013 Apr 5;8(4):e60178. doi: 10.1371/journal.pone.0060178 (PMC3618171; doi:10.1371/journal.pone.0060178)
Supplement: Methods S2 — Protocol for this meta-analysis. (DOCX) [file pone.0060178.s002.docx]

***hOGG1* Ser326Cys Polymorphism and Risk of Hepatocellular Carcinoma: A Meta-analysis**

Protocol for a gene-disease association study review

Wenjun Wang, Shuangsuo Dang^*^, Yaping Li, Mingzhu Sun, Xiaoli Jia, Rui Wang, Jingkun Liu

Department of Infectious Diseases, Second Affiliated Hospital of Medical School, Xi’an Jiaotong University, Xi’an, China

**Background**

Hepatocellular carcinoma (HCC) is the sixth most prevalent cancer and the third most frequent cause of cancer-related death worldwide [[1](#_ENREF_1)]. Well-established risk factors for HCC include chronic infection with hepatitis B virus (HBV) or hepatitis C virus (HCV), exposure to aflatoxin B1, male gender, drinking, smoking, non-alcoholic fatty liver disease and diabetes [2-6]. However, most individuals with these known environmental risk factors never develop HCC while many HCC cases develop among individuals without those known risk factors, suggesting that genetic factors also play an important role in hepatocellular carcinogenesis [[7](#_ENREF_7),[8](#_ENREF_8)].

Human 8-hydroxyguanine glycosylase 1 (hOGG1) is a DNA glycosylase enzyme responsible for the excision of 8-oxoguanine, a mutagenic base byproduct which occurs as a result of exposure to reactive oxygen [[9](#_ENREF_9)]. The *hOGG1* gene, located on chromosome 3p26.2, is composed of eight exons and seven introns. Polymorphisms in this gene may alter glycosylase function and an individual’s ability to repair damaged DNA, possibly resulting in genetic instability that can foster carcinogenesis [[9](#_ENREF_9)]. Among many polymorphisms identified in the *hOGG1* gene, much interest has been focused on the Ser326Cys (C>G) polymorphism (rs1052133). It is in exon 7 of the *hOGG1* gene, which takes the form of a single amino acid substitution, from serine to cysteine at condon 326. Although the evidence is inclusive that this functional polymorphic variation influences the activity of hOGG1 [[9](#_ENREF_9)], many epidemiologic studies have been conducted to examine its relationship with cancer risk.

We performed a presearch for studies on the association of the *hOGG1* Ser326Cys polymorphism and HCC risk. Several studies were identified. However, their results were inconsistent. No meta-analyses or systematic reviews have been published on this topic. Therefore, we plan to conduct a meta-analysis to clarify the inconsistency and to establish a comprehensive picture of this gene-disease association.

**Objectives**

To determine the association between the *hOGG1* Ser326Cys polymorphism and HCC risk.

**Methods**

**Criteria for considering studies:**

*Polymorphisms:*

Ser326Cys polymorphism in the *hOGG1* gene

*Health conditions:*

Hepatocellular carcinoma

*Types of study:*

Case-control study

**Search methods:**

Databases: PubMed, EMBASE, ISI Science Citation Index, BIOSIS, CNKI and WanFang;

Reference list of relevant articles;

Contact with authors for unpublished data.

Search terms: hepatocellular carcinoma, HCC, 8-Oxoguanine glycosylase, hOGG1

**Data extraction:**

Following the Meta-analysis Of Observational Studies in Epidemiology (MOOSE) statement for reporting meta-analyses of observational studies [10], we will use a standardized reporting form to abstract data from each included study. For each study, the following information will be extracted independently by two reviewers: the first author’s name, year of publication, study design, ethnicity, definition and numbers of cases and controls, confounding factors by matching or adjustment, genotyping method, frequency of genotypes, ORs and 95% CIs for HCC associated with the *hOGG1* Ser326Cys polymorphism, and consistency of genotype frequencies with Hardy-Weinberg equilibrium (HWE) in control subjects.

**Statistical analysis:**

Heterogeneity assumption will be checked by the *I^2^* statistic and a chi-square based *Q* test. A *P* value of more than 0.05 for the *Q* test indicates a lack of heterogeneity among the studies, so the summary OR estimate of each study will be calculated by the fixed-effect model (the Mantel-Haenszel method) [[11](#_ENREF_20)]. Otherwise, the random-effect model (DerSimonian and Laird method) will be used [[12](#_ENREF_21)]. Egger’s test and Begg’s graphical methods will be used to provide diagnosis of the potential publication bias [[13](#_ENREF_22)]. When data available, we will perform subgroup analysis and sensitivity analysis.

**Reference**

1. Ferlay J, Shin HR, Bray F, Forman D, Mathers C, et al. (2010) Estimates of worldwide burden of cancer in 2008: GLOBOCAN 2008. Int J Cancer 127: 2893-2917.

2. Donato F, Tagger A, Gelatti U, Parrinello G, Boffetta P, et al. (2002) Alcohol and hepatocellular carcinoma: the effect of lifetime intake and hepatitis virus infections in men and women. Am J Epidemiol 155: 323-331.

3. El-Serag HB (2011) Hepatocellular carcinoma. N Engl J Med 365: 1118-1127.

4. El-Serag HB, Tran T, Everhart JE (2004) Diabetes increase the risk of chronic liver disease and hepatocellular carcinoma. Gastroenterology 126: 460-468.

5. Forner A, Llovet JM, Bruix J (2012) Hepatocellular carcinoma. Lancet 379: 1245-1255.

6. Marrero JA, Fontana RJ, Fu S, Conjeevaram HS, Su GL, et al. (2005) Alcohol, tobacco and obesity are synergistic risk factors for hepatocellular carcinoma. J Hepatol 42: 218-224.

7. Dragani TA (2010) Risk of HCC: genetic heterogeneity and complex genetics. J Hepatol 52: 252-257.

8. Wang B, Huang G, Wang D, Li AJ, Xu ZP, et al. (2010) Null genotypes of GSTM1 and GSTT1 contribute to hepatocellular carcinoma risk: evidence from an updated meta-analysis. J Hepatol 53: 508-518.

9. Weiss JM, Goode EL, Ladiges WC, Ulrich CM (2005) Polymorphic variation in hOGG1 and risk of cancer: a review of the functional and epidemiologic literature. Mol Carcinog 42: 127-141.

10. Stroup DF, Berlin JA, Morton SC, Olkin I, Williamson GD, et al. (2000) Meta-analysis of observational studies in epidemiology: a proposal for reporting. JAMA 283: 2008-2012.

11. Mantel N, Haenszel W (1959) Statistical aspects of the analysis of data from retrospective studies of disease. J Natl Cancer Inst 22: 719-748.

12. DerSimonian R, Laird N (1986) Meta-analysis in clinical trials. Control Clin Trials 7: 177-188.

13. Egger M, Smith GD, Schneider M, Minder C (1997) Bias in meta-analysis detected by a simple, graphical test. BMJ 315: 629-634.
